# Genes, social transmission, but not maternal effects influence responses of wild Japanese macaques (*Macaca fuscata*) to novel-object and novel-food tests

**DOI:** 10.1007/s10329-016-0572-9

**Published:** 2016-09-12

**Authors:** Coline M. Arnaud, Takafumi Suzumura, Eiji Inoue, Mark J. Adams, Alexander Weiss, Miho Inoue-Murayama

**Affiliations:** 1 Wildlife Research Center of Kyoto University, Kyoto, Japan; 2 Faculty of Science, Toho University, Ota, Japan; 3 Department of Psychiatry, The University of Edinburgh, Edinburgh, UK; 4 Department of Psychology, School of Philosophy, Psychology and Language Sciences, The University of Edinburgh, 7 George Square, Edinburgh, EH8 9JZ UK

**Keywords:** Personality, Exploration, Inheritance, Genetics, Macaque

## Abstract

Using long-term maternal pedigree data, microsatellite analysis, and behavioral tests, we examined whether personality differences in wild Japanese macaques (*Macaca fuscata*) are associated with additive genetic effects, maternal influences, or belonging to a particular social group. Behaviors elicited by novel-object tests were defined by a component related to caution around novel-objects (Ob-PC1) and behaviors elicited by novel food-tests were defined by correlated components related to consummatory responses (Fo-PC1) and caution around novel foods (Fo-PC2). The repeatability of Ob-PC1 was modest and not significant; the repeatabilities of Fo-PC1 and Fo-PC2 were moderate and significant. Linear mixed effects models found that sex, age, sex × age, provisioning, trial number, date, time of day, season, and distance to the closest monkey were not related to personality. Linear mixed effects models of females older than 2 years found that high rank was associated with greater caution around novel objects. Linear models were used to determine whether sex, age, group membership, maternal kinship, or relatedness had independent effects on the personality similarity of dyads. These analyses found that pairs of macaques that lived in the same group were less similar in their caution around novel objects, more closely related pairs of macaques were more similar in their tendency to eat novel food, and that pairs of macaques in the same group were more similar in how cautious they were around novel foods. Together, these findings suggest that personality in this population of wild monkeys was driven by rank, genetic effects, and group effects, the latter possibly including the need to exploit different niches in the environment.

**Electronic supplementary material:**

The online version of this article (doi:10.1007/s10329-016-0572-9) contains supplementary material, which is available to authorized users.

## Introduction

Exploration is a personality trait observed in many species that describes individual differences in the tendency to approach or avoid novel situations (Réale et al. 2007). This personality trait is therefore analogous to the openness dimensions identified in several nonhuman primate taxa (King and Figueredo 1997; Weiss et al. 2011; Morton et al. 2013; Adams et al. 2015) and also in humans, where, in addition to being associated with curiosity and an interest in novel experiences, it is also associated with aesthetic sensitivities and liberal political values (McCrae and Costa 1997).

In addition to its wide distribution across species, exploration is related to longer survival (Smith and Blumstein 2008) and heritable (van Oers et al. 2005). However, because mechanisms apart from the transmission of additive genetic effects can cause relatives to resemble one another, if parental effects are not included in the models, the heritability of this trait might be overestimated (Wilson et al. 2010). Two alternatives to additive genetic effects are parental genetic and parental environment effects. An example of the former would be if some heritable behavioral or physiological phenotype on the part of the mother influenced the behavior of her offspring (Wolf and Wade 2009). An example of the latter would be if some environmentally influenced behavioral or physiological parental phenotype influences their offspring’s behavior. Another possible way in which resemblance among relatives may come about is via social transmission whereby offspring learn the behavior from observing one or both parents (Danchin and Wagner 2010; Danchin et al. 2011).

Cross-fostering designs have been used to distinguish between parental and genetic effects. For example, Bize et al. (2012) showed that anti-predator behavior in the alpine swift, *Apus melba*, is influenced by genetics and/or pre-hatching maternal effects. On the other hand, exploratory behavior in the zebra finch, *Taeniopygia guttata*, is not heritable, but is socially transmitted by parents (Schuett et al. 2013). As duration and type of parental care differ between species and taxa, the importance of social inheritance in the evolutionary processes may vary significantly. Thus, for many personality traits, in many species, the mode of inheritance remains unclear. To better understand the mode by which personality traits are transmitted, we measured personality traits related to a novel-object and a novel-food test and identified the bases of individual differences in these traits in wild Japanese macaques. As such, after testing for the possible effects of social rank and controlling for age and sex, we tested three competing hypotheses. The first hypothesis is that, as in the closely related rhesus macaques, *Macaca mulatta* (Brent et al. 2014), personality differences reflect additive genetic effects. The second hypothesis is that, because maternal care is long and dietary preferences and other traits are learned from the mother in Japanese macaques (Nakamichi and Yamada 2010), personality variation arises from maternal influences. The third hypothesis is that other forms of social transmission, such as learning from group members, which has been found in this population (Kawai 1965), can account for personality differences.

In the present study, to distinguished between additive genetic and maternal effects by using genetic markers to determine the relatedness among individuals, and identified both mother–offspring pairs and maternal sibling pairs. To distinguish between genetic and maternal effects and variation related to other forms of social transmission, we took advantage of the fact that the Japanese macaques in this study were either solitary or belonged to one of two social groups. Thus, for any given trait, we were able to use a multiple regression analysis to test the degree to which personality similarity among pairs of macaques is related to genetic relatedness, being maternal kin, and living in the same group.

## Methods

### Macaques and study site

We studied a population of Japanese macaques on Koshima, an islet located in southern Japan (31°N, 131°E, maximum elevation 114 m). This 32 ha islet is situated in a warm-temperate zone and is mainly covered by evergreen, broadleaved natural forests. This population has been monitored, occasionally trapped, and provisioned by technicians since 1952 (Iwamoto 1974; Watanabe 2001). Most of the macaques have thus been habituated to humans and can be approached up to a few meters. All macaques were individually recognizable by facial features and by small blue tattoo marks that some monkeys received during the last trapping season in 2007. Maternal lineages have been known since 1952.

Japanese macaques live in mixed-sex social groups characterized by female philopatry and a steep dominance hierarchy (Nakagawa 2010). The Koshima population comprises two social groups of 43 (main group) and 22 (Maki group) individuals, and 23 males live alone or in small groups of two or three individuals. Details about the social composition and age structure of the groups are given in Appendix S1. During the study period, 3 kg of common wheat were provided for the main group at the beach, in the morning, twice a week. The Maki group and solitary males were provisioned once a month and on rare occasions joined the regular provisioning of the main group.

Female social rank (low, middle, high) was based on observations of dominant (chasing another macaque) and submissive (being chased by another macaque) behaviors by TS, especially during provisioning. Seven female juveniles and sub-adults whose mothers were alive received the rank of their mother as social rank is socially inherited by the mother in Japanese macaques (Mori et al. 1989). It was not possible to estimate the rank of male juveniles and solitary males.

Experimental protocols were assessed and approved by the Animal Experiment Committee of Wildlife Research Center of Kyoto University.

### Personality assessment

We assessed personality in 70 macaques in the field during winter (February to mid-March) and spring (May to mid-June) of 2013. Macaques were testing using novel-object tests (*n* = 22), novel-food tests (*n* = 3), or both tests (*n* = 45). Each of the 67 macaques that took part in novel-object test was tested between 1 and 4 times (mean of 2.0 test trials per macaque) for a total of 136 test trials. Each of the 48 macaques that took part in the novel-food test was also tested between 1 and 4 times (mean of 2.5 test trials per macaque) for a total of 120 test trials.

**Fig. 1:**
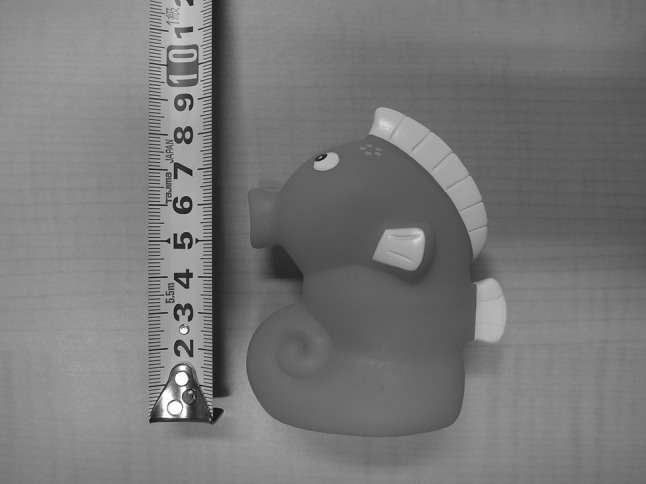
*Pink* plastic toy used as novel object (color figure online)

The novel-object test consisted of presenting an unfamiliar pink plastic toy measuring 85 mm × 70 mm × 35 mm (Fig. 1). If the toy was touched or manipulated, it was cleaned with a piece of kitchen towel soaked in a 70 % ethanol solution in water immediately after the trial ended. To reduce the potential effect of the ethanol odor during the next trial, for the 10 trials that were not the last of the day, we waited at least 5 min before beginning the next trial. The novel-food test consisted of presenting an unfamiliar food item, a boiled and peeled quail egg.

Individuals were tested when they were immobile on the ground in the forest (preferably sitting) and either foraging on the ground (mainly in winter) or looking for the next tree to forage, also called “visual-foraging” (mainly in spring). It was impossible to predict individual movements during foraging in the forest and few trails were used for group movements, so the novel object or food could not be placed ahead of time. In addition, the macaques did not attend to human objects, such as plastic bottles, that had washed up on shore or were lost by tourists.

Given these local conditions, we used the following testing procedure. The experimenter (CA) approached the focal individual up to 2.5–3 m and put the item on the ground, making sure that the monkey could see the item. The novel object, the unconsumed egg, or as many of the remaining pieces as possible were removed from the environment immediately after each trial. During data collection, the experimenter stayed at least 5 m away from the focal individual.

Tests were performed in isolated conditions as often as possible, and so we could not randomize the order of testing. The macaques we studied were those that visited the testing area during their daily travels. Care was taken to select monkeys so that there would be as little overlap as possible. To limit the influence of other monkeys, each individual was tested when no other individual was close by. This also prevented the possibility that other monkeys would experience the experimental stimuli before their own trial. To limit the possible influence of human tourists, testing took place away from the provisioned areas that are open to tourists and during times when tourists visited the field site.

Each trial lasted 150 s. Before each trial, the experimenter estimated the distance between the target monkey and the other monkeys. When no monkey could be seen on the ground or in the trees, the experimenter recorded a distance of 30 m. The mean distance between the focal individual and another individual at the beginning of a trial was about 16 m for the novel-object test and about 18 m for the novel-food test. The minimal distance allowed for the experiment to take place was 6 m when relief or landscape elements reduced other individuals’ visibility. In winter, one female subject had a 6-month-old infant that was becoming independent. She was tested when her infant was playing or foraging at least 4 m from her. In spring, adult females were tested before they gave birth.

Experiments were filmed to facilitate data extraction. CA coded the videos. Before CA coded the videos, she and an assistant independently coded 15 videos to determine which behaviors to extract, i.e., showed high levels of agreement and were thus easy to code reliably. There were seven such behaviors, all related to movements toward the item and inspection for both kinds of tests and seven variables related to manipulation and tasting during the novel-food test (Table 1).

**Table 1:** Behavioral variables recorded during the novel-object test and the novel-food test

Variable	Definition
Novel-object and novel-food test	
Moving latency	Latency to move towards the novel object or novel food item
Contact area latency	Latency to be close enough to be able to touch the novel object or novel food item. Distance estimated with the length of the trunk and half the length of the arm of the focal individual (0.5–1.0 m)
Interacting latency	Latency to interact with (smell, touch, or handle) the novel object or novel food item
Smelling duration	Total time spent smelling the novel object or novel food item
Touch	Number of times the novel object or novel food item was touched during the trial
Contact area duration	Total time spent in touching distance of the novel object or novel food item during the trial
Handling duration	Total time spent holding and manipulating the item
Novel-food test	
Handling latency	Latency to hold and manipulate the novel food item
Tasting latency	Latency to taste the novel food item
Taste	Total number of times during the trial the novel food item was tasted during the trial
Handling smell	Total number of times during the trial that the novel food item was smelled while being held and manipulated
Open	Whether the egg was separated it in two pieces during the trial
Dissect	Whether the egg was separated into small pieces during the trial
Consume	Rough proportion of the egg that was eaten during the trial

Durations recorded in seconds. Latencies recorded as seconds from the beginning of the trial and received a maximum value of 150 s if the subject never showed the behavior

### Microsatellite analyses

Fecal samples were collected opportunistically during the twice-a-week provisioning of the main group and during the monthly provisioning and weighing of individuals in the Maki group and solitary males. Weighing took place during a routine health check and was non-invasive in that macaques were rewarded for standing on a scale with a small food reward.

DNA was extracted using a QIAamp DNA Stool Mini Kit (Qiagen, CA, USA). We used 16 microsatellite loci (10 tetra-nucleotide repeats and six di-nucleotide repeats; Inoue and Takenaka 1993; Domingo-Roura et al. 1997; Inoue and Takenaka 2008; Kawamoto et al. 2008). Further details on genetic work are provided in the Supplemental Methods. Based on these 16 microsatellite loci containing 92 alleles, we used COANCESTRY version 1.0.1.2 (Wang 2011) to estimate the Queller and Goodnight (1989) relatedness index, which ranges between −1 and 1, for 99 genotyped monkeys including some deceased individuals. Mean relatedness was −0.009 ± 0.003 SE. with a range from −0.51 to 0.89.

### Statistical analyses

All analyses were conducted using version 3.3.1 of R (R Core Team 2016). We first reduced the number of behavioral variables by using the principal function (Revelle 2015) to conduct principal components analyses (PCA). For these analyses we used the complete data on behaviors recorded during novel-object tests (67 macaques and 136 trials) and during novel-food tests (48 macaques and 120 trials). To determine how many components to retain for each set of behavioral variables, we inspected the scree plot and used the fa.parallel function (Revelle 2015) to conduct parallel analyses (Horn 1965). We rotated any factors using an orthogonal (varimax) and oblique (promax) rotation. We then obtained differentially weighted (regression method) component scores and used these scores in all further analyses.

Next, for each component derived in our PCAs, we used lmer (Bates et al. 2015) to fit two sets of linear mixed effects models via a restricted maximum likelihood estimation procedure. For components related to novel-object tests, complete data were only available for 63 macaques (123 tests).

The first set of linear mixed effects models included monkey identity as a random effect and sex, age, sex × age, trial, date, time of day, season (winter or spring), provisioning, and distance to the closest monkey when the trial began as fixed effects. We did not include social group in these analyses because sample sizes were small for the Maki group (*n* = 12 for novel-object tests; *n* = 14 for novel-food tests). However, as social groups in Koshima share a similar environment with overlapping home ranges and were regularly mixed due to migration flows (Yamagiwa 2010), it is unlikely that group effects would influence our results. Similarly, because of sample size limitations, we did not test for sex × season interaction effects. In these analyses, we obtained *P* values based on *t* tests and Satterthwaite approximations of degrees of freedom (Kuznetsova et al. 2016).

The second set of linear mixed effects models included monkey identity as a random effect and rank (high, middle, and low) as a fixed effect. We restricted these analyses to females who were more than 2 years old because younger females would still be spending most of their time with their mother. These analyses were thus based on 66 novel-object tests carried out on 30 females (10 high-, 10 middle-, and 10 low-ranking) and 75 novel-food tests carried out on 26 females (seven high-, 10 middle-, and nine low-ranking). In these analyses, we determined whether the overall effect of rank was significant by conducting an *F* test with Satterthwaite approximations of degrees of freedom (Kuznetsova et al. 2016). We then used the glht function of the multcomp package (Hothorn et al. 2008) to conduct post hoc Tukey’s honest significant difference tests to compare the low, average, and high ranking macaques. The *P* values in the post hoc tests were adjusted for multiple testing using the single-step method (Hothorn et al. 2008).

We then used the rptR package (Schielzeth and Nakagawa 2013) to estimate the repeatabilities of the components by fitting linear mixed effects models with monkey identity as a random effect (Eq. 11 in Nakagawa and Schielzeth 2010). We estimated 95 % confidence intervals (CI) by means of parametric bootstrapping (Schielzeth and Nakagawa 2013). We did not include sex, age, sex × age, trial, date, time of day, season, provisioning, or distance to the closest monkey as fixed effects since none were significant in the previous analyses. Furthermore, for the novel-object tests and novel-food tests we restricted these analyses to 43 macaques (112 tests) and 36 macaques (108 tests) that were tested on more than one occasion, respectively.

Next, in 46 macaques tested at least once with the novel-object test and at least once with the novel-food test, we obtained mean individual component scores across all trials and computed the correlations between novel-object components and novel-food components. Because none of the fixed effects were significant, we did not use residuals or best linear unbiased predictors from the mixed models to control for fixed effects.

Because our sample size was small and DNA samples were missing for up to 15 candidate sires, we could not complete the pedigree. We were thus unable to obtain the variance components used to compute additive genetic (heritability) or maternal effect estimates from parent-offspring regressions (Falconer and Mackay 1996) or animal models (Wilson et al. 2010). Consequently, we used an alternative approach to investigate the influence of genes, maternal kinship, and group membership on personality differences in these macaques. Our approach involved first computing, for each component, the absolute difference in component scores between members of all possible dyads. We first, for each component, regressed the difference score on the Queller and Goodnight relatedness index. We then, again for each component, regressed the difference score onto the Queller and Goodnight relatedness index and variables indicating whether it was a same sex dyad (0 = no, 1 = yes), the absolute difference in age between the macaques (in years), whether the macaques belonged to the same group (0 = no, 1 = yes), and whether the macaques were either maternal siblings or a mother-offspring pair (0 = no, 1 = yes). Because there is some non-independence in these analyses (individuals are repeated across dyads) and the unit of analysis is not the individual but the dyad, instead of including random effects of individuals we used the lm.boot function (Peng 2008) to obtain bootstrapped 95 % confidence intervals.

## Results

We provide more information about sex, age, and social group in Appendix S2. For the novel-object test and the novel-food test, 58 and 19 % of the individuals, respectively, never approached the item and continued their own activity during the whole trial.

For the novel-object test, we retained one component (Ob-PC1) that explained 53 % of the variance (Table 2). This component appeared to be related to caution around a novel object as higher levels were associated with taking longer to approach and interact with the object, spending less time smelling the object and being in the proximity of the object, and touching the object less frequently. For the novel-food test we retained two oblique (*r* = −0.41) components (Fo-PC1 and Fo-PC2) that explained 68 % of the variance (Table 3). The first component appeared to be related to consummatory responses as higher levels were associated with spending more time near eggs, taking less time to handle and taste eggs, and opening, dissecting, and eating the eggs. The second component appeared to be related to caution around the novel food as higher levels were associated with taking more time to approach the egg and to interact with it, spending less time smelling the egg, and to be less likely to touch the egg. Caution around novel objects (Ob-PC1) was significantly correlated with novel around novel food items Fo-PC2 (*r* = 0.50, *P* < 0.001) but not with Fo-PC1 (*r* = −0.23, *P* = 0.14).

**Table 2:** Component loadings of novel-object test variables

	Ob-PC1
Contact area latency	**0.92**
Interacting latency	**0.91**
Smelling duration	**−0.80**
Moving latency	**0.75**
Contact area duration	**−0.65**
Touch	**−0.59**
Handling duration	−0.26

Component loadings greater than |0.4| in boldface

**Table 3:** Component loadings of novel-food test variables

	Fo-PC1	Fo-PC2
Open	**0.94**	0.06
Dissect	**0.90**	0.09
Handling duration	**0.88**	0.01
Taste	**0.82**	0.10
Tasting latency	**−0.81**	0.06
Contact area duration	**0.74**	−0.20
Handling smell	**0.73**	−0.12
Consume	**0.68**	0.06
Handling latency	**−0.60**	0.37
Contact area latency	−0.12	**0.90**
Interacting latency	−0.14	**0.89**
Moving latency	−0.08	**0.88**
Smelling duration	−0.34	**−0.85**
Touch	−0.09	**−0.40**
Proportion of variance	0.43	0.25

Component loadings greater than |0.4| in boldface

The repeatability of caution around novel objects was modest and not significant [Ob-PC1: R ± SE = 0.12 ± 0.10, 95 % CI = (0.00, 0.33), *P* = 0.12]. The repeatability of consuming the novel food and caution around the novel food were moderate and significant [Fo-PC1: R ± SE = 0.39 ± 0.11, 95 % CI = (0.15, 0.58), *P* = 0.002; Fo-PC2: R ± SE = 0.31 ± 0.11, 95 % CI = (0.08, 0.52), *P* = 0.001].

Sex, age, provisioning during the test day, trial number, date, time of day, season, sex × age, and distance to the closest monkey were not significantly associated with the novel object component or the novel-food test components (Table 4). For the novel-object test, 80 % of high ranking females (versus 60 % of the medium and low ranking females) never approached the novel object and continued their own activity during the whole trial. For Ob-PC1, the overall effect of rank was not significant (*F*_2,10.642_ = 3.86, *P* = 0.055). Post-hoc tests revealed that the difference between low and high ranking females was significant (ΔY_low–high_ ± SE = −0.73 ± 0.30, *t* = −2.41, *P* = 0.042) and that neither the difference between middle and high ranking females (ΔY_middle–high_ ± SE = −0.66 ± 0.28, *t* = −2.32, *P* = 0.053) or between low and middle ranking females were (ΔY_low–middle_ ± SE = −0.07 ± 0.31, *t* = −0.23, *P* = 0.97) significant. The overall effect of rank on Fo-PC1 was not significant (*F*_2,26.653_ = 0.97, *P* = 0.39). Post-hoc tests revealed that neither the difference between middle and high ranking females (ΔY_middle–high_ ± SE = 0.55 ± 0.40, *t* = 1.37, *P* = 0.36), the difference between low and high ranking females (ΔY_low–high_ ± SE = 0.41 ± 0.41, *t* = 1.01, *P* = 0.57), nor the difference between low and middle ranking females were (ΔY_low–middle_ ± SE = −0.13 ± 0.36, *t* = −0.37, *P* = 0.93) significant. The overall effect of rank on Fo-PC2 was not significant (*F*_2,21.112_ = 0.43, *P* = 0.66). Post-hoc tests revealed that neither the difference between middle and high ranking females (ΔY_middle–high_ ± SE = −0.33 ± 0.41, *t* = −0.81, *P* = 0.69), the difference between low and high ranking females (ΔY_low–high_ ± s.e. = −0.36 ± 0.42, *t* = −0.84, *P* = 0.68), nor the difference between low and middle ranking females were (ΔY_low–middle_ ± SE = −0.02 ± 0.37, *t* = −0.06, *P* > 0.99) significant.

**Table 4:** Estimates of fixed effects from a linear mixed models with monkey identity as random effect

Fixed effect	Component				
	*b*	SE	*df*	*t*	*P*
Ob-PC1					
Intercept	1.21	1.90	104.12	0.64	0.52
Sex*	−0.45	0.54	38.12	−0.83	0.41
Age	0.01	0.04	40.35	0.29	0.77
Provisioning^†^	−0.10	0.17	70.85	−0.56	0.58
Trial number	0.18	0.12	107.76	1.51	0.13
Date	−0.01	0.01	112.57	−1.02	0.31
Time	0.00	0.04	87.73	−0.10	0.92
Season^‡^	−0.78	1.08	107.28	−0.72	0.47
Sex × Age	−0.01	0.01	88.41	−0.78	0.43
Distance	0.06	0.06	39.76	1.11	0.27
Fo-PC1					
Intercept	−4.34	3.11	107.13	−1.40	0.17
Sex*	−0.46	0.47	52.31	−0.97	0.33
Age	−0.04	0.03	44.69	−1.34	0.19
Provisioning^†^	0.09	0.18	96.13	0.48	0.64
Trial number	0.06	0.10	106.48	0.62	0.54
Date	0.02	0.02	108.50	1.26	0.21
Time	0.02	0.04	103.29	0.50	0.62
Season^‡^	2.85	1.94	106.36	1.47	0.14
Sex × Age	0.01	0.01	107.17	1.00	0.32
Distance	0.01	0.06	50.21	0.18	0.86
Fo-PC2					
Intercept	−4.51	3.35	105.63	−1.35	0.18
Sex*	0.23	0.49	34.93	0.46	0.65
Age	0.05	0.03	28.48	1.42	0.17
Provisioning^†^	−0.26	0.19	87.21	−1.36	0.18
Trial number	−0.03	0.11	102.63	−0.23	0.82
Date	0.02	0.02	107.90	1.10	0.27
Time	0.06	0.04	99.14	1.28	0.20
Season^‡^	2.07	2.09	104.38	0.99	0.32
Sex × Age	−0.01	0.01	105.89	−0.69	0.49
Distance	−0.01	0.06	32.89	−0.25	0.81

Dependent variables were standardized (mean = 0, SD = 1) for the analyses

* Female was the reference category

^†^No provisioning occurring the day of the trial was the reference category

^‡^Spring was the reference for category

For the macaques in the novel-object tests, relatedness data were available for 1485 dyads. The median relatedness of the 1436 unrelated pairs was −0.03 (range −0.49 to 0.89). The median relatedness of the 34 maternal sibling pairs was 0.17 (range −0.12 to 0.54). The median relatedness of the 17 mother-offspring pairs was 0.46 (range 0.28–0.58). In the unadjusted and fully-adjusted models, relatedness was not associated with differences in Ob-PC1; however, in the adjusted model there was a significant effect of group such that pairs within the same group differed more from one another than pairs that were not in the same group (Table 5).

**Table 5:** Dyad similarity in personality as a function of additive genetic effects, maternal effects, and sex, age, and group membership effects

	*b*	SE	*t*	*P*	95 % CI	
					2.50 %	97.50 %
Ob-PC1						
Unadjusted						
Intercept	1.04	0.03	34.53	<0.001	0.98	1.10
*R*_Q_	−0.07	0.17	−0.45	0.65	−0.38	0.25
Fully-adjusted						
Intercept	0.96	0.07	13.52	<0.001	0.83	1.09
Sex*	−0.04	0.06	−0.75	0.45	−0.16	0.07
Age	0.00	0.01	0.02	0.98	−0.02	0.02
Group^†^	**0.15**	**0.06**	**2.36**	**0.018**	**0.03**	**0.27**
Maternal kinship^‡^	0.11	0.18	0.65	0.52	−0.26	0.52
*R*_Q_	−0.09	0.17	−0.49	0.62	−0.42	0.26
Fo-PC1						
Unadjusted						
Intercept	0.61	0.03	23.60	<0.001	0.56	0.66
*R*_Q_	**−0.34**	**0.15**	**−2.35**	**0.019**	**−0.63**	**−0.06**
Fully-adjusted						
Intercept	0.55	0.12	4.48	<0.001	0.42	0.72
Sex*	0.10	0.05	1.87	0.062	−0.01	0.20
Age	0.00	0.01	0.54	0.59	−0.01	0.02
Group^†^	−0.01	0.12	−0.09	0.93	−0.17	0.12
Maternal kinship^‡^	−0.02	0.14	−0.18	0.86	−0.23	0.22
*R*_Q_	**−0.34**	**0.15**	**−2.17**	**0.030**	**−0.63**	**−0.05**
Fo-PC2						
Unadjusted						
Intercept	1.00	0.02	40.53	<0.001	0.95	1.05
*R*	0.06	0.14	0.44	0.66	−0.21	0.33
Fully-adjusted						
Intercept	1.23	0.12	10.53	<0.001	1.00	1.47
Sex*	−0.05	0.05	−0.95	0.34	−0.14	0.05
Age	0.00	0.01	0.61	0.54	−0.01	0.02
Group^†^	**−0.24**	**0.12**	**−2.05**	**0.041**	**−0.47**	**−0.01**
Maternal kinship^‡^	−0.04	0.13	−0.28	0.78	−0.34	0.29
*R*_Q_	0.07	0.15	0.49	0.63	−0.21	0.35

Significant effects, i.e., those with confidence intervals that do not include zero, are in boldface

95 % CI = bootstrapped 95 % confidence intervals generated by resampling rows 5000 times

Age = Effect of absolute difference in age

* Same-sex is the reference category

^†^Not being in the same group is the reference category

^‡^Not being a mother-offspring pair or maternal siblings is the reference category

For the macaques in the novel-food tests, relatedness data were available for 903 dyads. The median relatedness of the 864 unrelated pairs was −0.05 (range −0.49 to 0.66). The median relatedness of the 24 maternal sibling pairs was 0.15 (range −0.12 to 0.54). The median relatedness of the 15 mother-offspring pairs was 0.46 (range 0.28–0.58). In the unadjusted and fully-adjusted models, relatedness was associated with greater pair similarity in Fo-PC1; no other effects were significant (Table 5). In the unadjusted and fully adjusted models, relatedness was not associated with differences in Fo-PC2; however, in the adjusted model there was a significant effect of group such that pairs within the same group were more similar to one another than pairs that were not in the same group (Table 5).

## Discussion

In wild Japanese macaques, we found that one component described behavioral responses towards a novel object and two components described behavioral responses towards a novel food. We also found that macaques were consistent in how they reacted to novel-food tests, but not to the novel-object tests. However, the component that described the macaques’ behavior in novel-object tests was positively correlated with a component describing the time it took for them move toward and interact with the novel food. In addition to these findings, we found that high ranking females were more cautious around novel objects than middle or low ranking females and that genetic effects were associated with a tendency to eat the novel food and being in the same group was related to individuals being more different and more similar in their degree of caution around novel objects and novel food items, respectively.

The components related to behaviors evoked by the novel-food tests resembled components related to behaviors evoked by chacma baboons, *Papio ursinus*, in response to similar tests (Carter et al. 2012). Moreover, the repeatability for these components were comparable to the repeatabilities found in studies of other species (Bell et al. 2009). On the other hand, the repeatability for the novel-object test component was low compared to the repeatabilities of personality traits found in studies of other studies (Bell et al. 2009). One possible explanation for this difference in repeatability is that the variability in responses to novel-object tests was too low. This is consistent with the fact that more than half of the macaques never approached the novel object whereas only 18 % of the macaques never approached the novel food. This difference in the variability may be because the smell of our novel object might be similar to non-edible plastic objects, such as plastic bottles, that the macaques previously encountered. On the other hand, some Koshima macaques have encountered and eaten the eggs of wild birds (personal observation). However, this behavior is rare and, suffice it to say, these macaques would never have encountered eggs that were boiled and peeled. Still, the quail eggs that we used might have smelled or looked similar to eggs the macaques encountered and thus evoked more interest than the toy. Alternatively, because this population is not under high predation pressure, novel food items might be a better means to assess exploration than novel objects.

Our finding that high ranking females were more cautious around the novel object than are middle and low ranking females is not consistent with observations in other nonhuman primate species. For example, a review of the primate literature by Clarke and Boinski (1995) found that subordinate individuals tended to be shyer and more fearful than dominant individuals. Likewise, studies of several macaque species, including Japanese macaques, find that macaques that are higher in personality dimensions related to dominance, assertiveness, or confidence tend to be rated as less timid, shy, and fearful (Stevenson-Hinde and Zunz 1978; Capitanio and Widaman 2005; Weiss et al. 2011; Adams et al. 2015). The most likely explanation for our seemingly contradictory results, then, is that the measures used in previous studies were closer to boldness than to exploration. This is consistent with findings from a study of captive chimpanzees, *Pan troglodytes*, that showed no effect of social rank on exploratory behaviors (Massen et al. 2013) and of a meta-analysis that found evidence for only a weak association between exploration and aggression in several species (Garamszegi et al. 2013). This meta-analysis also pointed out that the correlation differed between novel-environment and novel-object tests, suggesting that different novel stimuli and/or situations may be measures of different constructs, see, e.g., a recent study of mountain chickadees, *Poecile gambeli* (Fox et al. 2009). Moreover, a study of personality ratings in multiple macaque species consistently found that ratings on traits related to exploratory behaviors are mostly unrelated to personality dimensions associated with dominance, confidence, and assertiveness (Adams et al. 2015).

Our finding that responses to novel food were not associated with rank runs counter to the fact that Japanese macaques compete over access to food. Studies of provisioned Japanese macaques have found, for example, that high ranking females obtain their energy mainly from artificial food whereas lower ranking females gain energy from natural food (Soumah and Yokota 1991). Also, as we noted earlier, access to food is used to measure rank in this population. One possibility is that these rank-related differences in gaining access to food are limited to familiar foods and not novel foods. Another possibility is that the effect of rank only holds in group settings and not when individuals are tested alone, as they were in the present study. Given the differences in methods between our study and other studies, it is too early to draw strong conclusions concerning why some of our findings appear to differ from other studies, and so we advise caution in interpreting these results. To gain a better understanding of these constructs and their associations with rank requires conducting individual and group tests involving different novel objects and situations.

Our finding that the tendency to eat a novel food is more similar between related individuals supports the hypothesis that this personality construct is genetically transmitted. This finding is consistent with those from heritability studies of nonhuman primates. For example, exploratory behaviors were heritable in infant rhesus macaques confronted with a novel food (Williamson et al. 2003) or with a novel-environment (Fawcett et al. 2014). Boldness in a social context was also found to be heritable in free-ranging rhesus macaques (Brent et al. 2014). Likewise, exploratory behavior in vervet monkeys, *Chlorocebus pygerythrus*, confronted with a novel object was heritable and associated with the dopamine receptor D4 genotype (Bailey et al. 2007).

On the other hand, the absence of maternal effects suggests that, although dietary preferences appear to be learned from the mother in Japanese macaques (Nakamichi and Yamada 2010), this may not extend to the tendency to consume novel foods. Alternatively, the similarity between mother and offspring or among maternal siblings in the tendency to eat novel foods is attributable to additive genetic effects. Previous studies of the heritability of nonhuman primate personality using animal models also have not found evidence for maternal effects (Weiss et al. 2002; Fairbanks et al. 2004; Adams et al. 2012; Brent et al. 2014). However, studies using an animal model (Wilson et al. 2010) that estimates the importance of genes, maternal effects, and environmental effects are needed to determine just how much genetic and non-genetic influences are responsible for variation in personality traits in Japanese macaques. Still, because the models in our study included maternal kinship and group environment effects, the present findings should at least partly control for maternal and group effects. The absence of a significant effect of group suggests similarity between group members arises because the home ranges of social groups in Koshima largely overlap (Yamagiwa 2010), which leads the two groups to be related.

We also found evidence that social transmission influenced caution around novel food items and that being in the same group leads to differences in how cautious individuals are around novel objects. While parental effects can help offspring “tune” their behavior to current environmental conditions (Reddon 2012), these effects can contribute to non-genetically transmitted variation. Potential mechanisms of non-genetic transmission include heritable epigenetic modifications (Ledon-Rettig et al. 2013), parental genetic and environmental effects, and social transmission via learning or ecological inheritance (Bonduriansky and Day 2009, Danchin et al. 2011). In addition, we were surprised that individuals within the same group were more different with respect to their tendency to be cautious around the novel object. This finding suggests some mechanism, e.g., early imprinting or plasticity, may lead individuals to adopt a strategy that differs from fellow group members, perhaps so that they can exploit niches that are not already occupied (Plomin and Daniels 1987; Stamps and Groothuis 2010). Future longitudinal studies should examine the bases of these effects. For example, the degree to which individuals modify how cautious they are around novel objects when they emigrate or when their group changes may index the degree to which these traits reflect early learning or are a response to the social environment.

One limitation of the present study is its relatively small size. This may explain why we did not find a significant genetic basis for the other two personality components or any maternal effects. Another limitation of the present study is that, because related females hold similar ranks, and rank data were not available for all of the females, we could not test whether our genetic influences would still be significant after including rank in the models. Finally, the fact that responses to the novel-object tests were not repeatable suggests that either the behaviors measured were not indicators of a latent construct, such as a personality trait, and the results related to these behaviors should be interpreted with caution.

Disentangling the genetic and social transmission of behavior in wild animal populations is a challenge for behavioral ecologists, particularly as both kinds of transmission can be important evolutionary forces (Danchin et al. 2011). For instance, according to Danchin (2013), it is possible for natural selection to act on a trait for which its variation is only socially transmitted and thus this trait would evolve without underlying genetic variation over the course of a few generations (short-term micro-evolution). Other authors have highlighted the importance of considering all types of transmitted variation in the evolutionary processes (Mameli 2004; Day and Bonduriansky 2011). Although larger, future samples with complete pedigrees are still needed, by distinguishing between genetic, maternal, and social transmission of personality traits in wild nonhuman primates, the present study represents an advance in our understanding of the evolution of personality in primates and other species.

## Electronic supplementary material

Below is the link to the electronic supplementary material.

Supplementary material 1 (DOCX 29 kb)
