# Supplementary material for: Genes, social transmission, but not maternal effects influence responses of wild Japanese macaques (Macaca fuscata) to novel-object and novel-food tests
Source: Primates. 2016 Sep 12;58(1):103–13. doi: 10.1007/s10329-016-0572-9 (PMC5215262; doi:10.1007/s10329-016-0572-9)
Supplement: Supplementary file 1 — Supplementary material 1 (DOCX 29 kb) [file 10329_2016_572_MOESM1_ESM.docx]

**Electronic Supplementary Materials**

**Supplementary Methods**

We performed multiplex PCR (Multiplex PCR kit, Qiagen) using the following settings: initial step at 95°C for 15 minutes, 45 cycles of denaturation at 94°C for 30 seconds, annealing at 57°C for 30 seconds, extension at 72°C for 30 seconds and final extension step at 60°C for 30 minutes. The first multiplex set included D3s1768, D5s820, D6s501, D17s1290, MFGT18, and MFGT22. The second multiplex set included D6s493, D14s306, D19s582, MFGT5, and MFGT21. The third multiplex set included D1s548, D7s821, D20s484, MFGT24, and MFGT27. Amplification products were separated by capillary electrophoresis using 3130xl Genetic Analyzer (Applied Biosystems, California, USA) with a molecular size standard (GeneScan 400HD ROX Size Standard, Applied Biosystems). GeneScan v. 4.0 and PeakScanner v. 1.0 were used to size the alleles. Heterozygous and homozygous loci were confirmed by at least two and three independent PCR runs, respectively.

**Appendix S1.** Social and age composition of the current Japanese macaque population of Koshima island.

|  |  | Adult males | Adult females | Juveniles and sub-adults | Total |
| --- | --- | --- | --- | --- | --- |
| Main group | Number | 5 | 20 | 18 | 43 |
|  | Mean age | 10.4 [7-18] | 11.4 [7-17] | 4.4 [2-6] |  |
| Maki group | Number | 3 | 7 | 12 | 22 |
|  | Mean age | 15.0 [12-18] | 14.6 [9-19] | 4.0 [2-6] |  |
| Solitary males | Number | 21 |  | 2 | 23 |
|  | Mean age | 10.4 [7-16] |  | 6 [6] |  |
| Total |  | 29 | 27 | 32 | 88 |

Age expressed in years. Ranges are in square brackets.

**Appendix S2.** Number, mean age, and age range of tested Japanese macaques in each social group and for each sex and age class.

|  |  | Adult males | Adult females | Juveniles and sub-adults | Total |
| --- | --- | --- | --- | --- | --- |
| NOVEL-OBJECT TEST | | | | | |
| Main group | Number | 4 | 20 | 16 | 40 |
|  | Mean age | 11.3 [7-18] | 11.4 [7-17] | 4.6 [2-6] |  |
| Maki group | Number | 2 | 4 | 2 | 8 |
|  | Mean age | 16.5 [15-18] | 15 [11-19] | 3.5 [2-5] |  |
| Solitary males | Number | 17 |  | 2 | 19 |
|  | Mean age | 10.5 [7-16] |  | 6 [6] |  |
| NOVEL-FOOD TEST | | | | | |
| Main group | Number | 5 | 20 | 17 | 42 |
|  | Mean age | 10.4 [7-18] | 11.4 [7-17] | 4.6 [2-6] |  |
| Maki group | Number | 1 |  |  | 1 |
|  | Mean age | 18 [18] |  |  |  |
| Solitary males | Number | 3 |  | 2 | 5 |
|  | Mean age | 8 [7-9] |  | 6 [6] |  |

Age expressed in years. Ranges are in square brackets.
